# Crystal structure of di-μ-hydroxido-bis­[aqua­(1,10-phenanthroline-κ^2^ *N*,*N*′)copper(II)] naphthalene-2,6-di­carboxyl­ate hexa­hydrate

**DOI:** 10.1107/S2056989015004338

**Published:** 2015-03-14

**Authors:** Daniela Arias-Zárate, María Fernanda Ballesteros-Rivas, Rubén A. Toscano, Jesús Valdés-Martínez

**Affiliations:** a UVM Campus Toluca, Avenida las Palmas Poniente No. 439 San Jorge Pueblo Nuevo, CP 52164, Metepec, Estado de México, Mexico; b Instituto de Química, Universidad Nacional Autónoma de México, Circuito Exterior s/n, Ciudad Universitaria, Cd. México, 04510, Coyoacán, México D.F., Mexico

**Keywords:** crystal structure, binuclear copper(II) complex, crystal engineering, hydrogen bonding, π–π stacking

## Abstract

In the title compound, [Cu_2_(OH)_2_(C_12_H_8_N_2_)_2_(H_2_O)_2_](C_12_H_6_O_4_)·6H_2_O, the two hydroxide groups bridge the two Cu^II^ cations, forming a centrosymmetric binuclear complex cation, in which the Cu^II^ cation is coordinated by a 1,10-phenanthroline (phen) mol­ecule, one water mol­ecule and two bridging hydroxide O atoms in a distorted N_2_O_3_ square-pyramidal coordination geometry. The naphthalene-2,6-di­carboxyl­ate anion is also located on an inversion centre. In the crystal, O—H⋯O hydrogen bonds link the cations, anions and lattice water mol­ecules into a three-dimensional supra­molecular architecture. Extensive π–π stacking is observed between the parallel or nearly parallel aromatic rings of adjacent phen ligands and naphthalenedi­carboxyl­ate anions, the centroid-to-centroid distances ranging from 3.4990 (16) to 3.8895 (16) Å.

## Abstract 2

The cations and anions of the title compound are organized through π–π stacking between the aromatic rings of the 1,10-phenanthroline and the naphthalene-2,6-carboxyl­ate into a two-dimensional structure. The extensive O—H⋯H hydrogen bonds further connect the cations, anions and lattice water mol­ecules into a three-dimensional network.

## Chemical context   

The designed arrangement of mol­ecules through inter­molecular inter­actions is one of the main purposes of crystal engineering. Among these inter­actions are hydrogen bonds and π–π stacking (Hunter & Sanders, 1990 ▸). π–π stacking inter­actions are ubiquitous in biological systems, and organic mol­ecules (Riley & Hobza, 2013 ▸; Klärner & Schrader, 2013 ▸), and are present in many metal complexes (Janiak, 2000 ▸). Nevertheless, relatively few systems have been designed to be organized mainly by π–π inter­actions (Putta *et al.*, 2014 ▸; Sebaoun *et al.*, 2014 ▸; Valdés-Martínez *et al.*, 2005 ▸). In most cases, they are secondary inter­actions helping to stabilize the network, not the main tool in the organization of the mol­ecules in the crystal. We have proved that it is possible to obtain designed non-centrosymmetric crystals through π–π stacking inter­actions (Serrano-Becerra *et al.*, 2009 ▸).
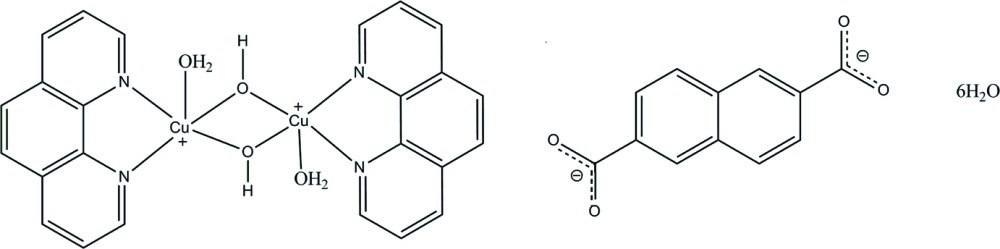


As part of a systematic study of the possible organization of copper coordination compounds controlled by π–π stacking inter­actions, we decided to use aromatic amines, as blocking ligands, and naphthalene-2,6-di­carboxyl­ate as a possible bridging ligand between the [Cu(ammine)] moieties, as long as all of them may form π–π inter­actions. The reactions were done in water – the tendency of carboxyl­ates to form hydrogen bonds with water is well known, as is their tendency to coord­inate to Cu^II^ complexes – so these structures will give us an opportunity to evaluate the importance of water⋯ hydrogen bonding *versus* π–π inter­actions as the main inter­action controlling the organization of the mol­ecules in the crystal.

During these studies, the title compound was unexpectedly obtained. Its mol­ecular and crystal structure are described herein.

## Structural commentary   

**Figure 1:**
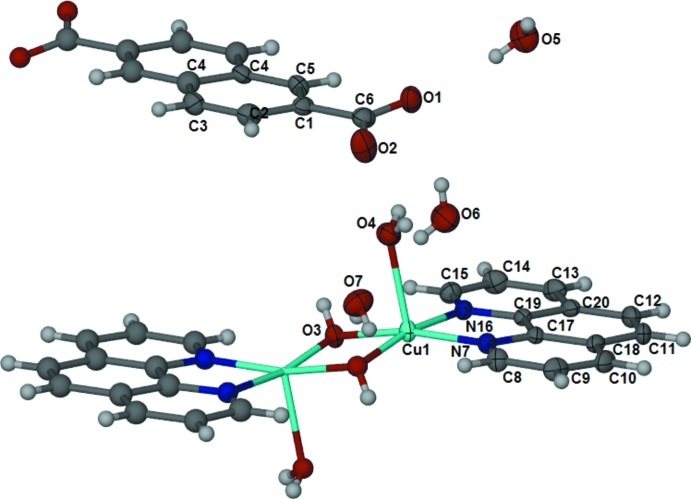
The structure of the title compound showing the atom-labelling scheme. Displacement ellipsoids are drawn at the 50% probability level. H atoms are shown as circles of arbitrary radius.

The asymmetric unit of the title compound contains half of a [(phen)(H_2_O)Cu(OH)_2_Cu(H_2_O)(phen)] (phen is 1,10-phen­anthroline) dimer, half of an naphthalene-2,6-di­carboxyl­ate anion and three lattice water mol­ecules. The Cu^II^ cation is penta­coordinated with a square-pyramidal geometry, the phen coordinates as a bidentate ligand through the N atoms, the hydroxide groups bridge the two Cu^II^ cations and a water mol­ecule is coordinated in the apical position (Fig. 1 ▸). The carboxyl­ate group of the naphthalene-2,6-di­carboxyl­ate anion is twisted at 12.4 (3)° with respect to the naphthalene ring system.

## Supra­molecular features   

**Figure 2:**
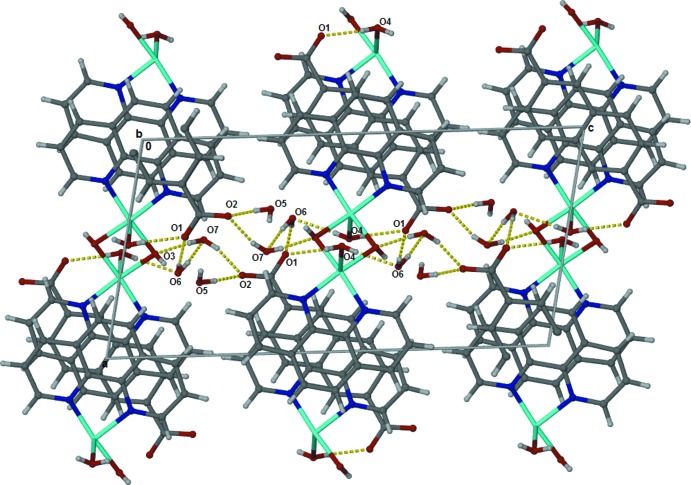
Crystal structure of the title compound viewed along the *b* axis, showing the hydrogen bonding, as dashed lines, and π–π stacking.

An extensive network of hydrogen bonds is formed (Table 1 ▸) in the crystal. Atom O4 of the coordinating water mol­ecule acts as a hydrogen-bond donor to O6 of a water mol­ecule and carboxyl­ate atom O1. The bridging hydroxide group hydrogen bonds to atom O5 of a water mol­ecule and acts as a hydrogen-bond acceptor with water oxygen atom O7. The carboxyl­ate atom O1 forms three hydrogen bonds while carboxyl­ate atom O2 forms two hydrogen bonds. Water oxygen atoms O6 and O7 form hydrogen bonds with each other as well as with the carboxyl­ate O atoms. The hydrogen-bond network extends into a three-dimensional structure, see Fig. 2 ▸. The presence of a free naphthalene-2,6-di­carboxyl­ate with four hydrogen-bond acceptors requires the presence of water mol­ecules, but the tendency of the aromatic rings in the ligands to form inter­actions may also observed and this is an important factor in the organization of the mol­ecules in the crystal (Fig. 2 ▸). Two phenanthroline units from two adjacent cations lie parallel, on top of each other, the distance between the centroids of the ligand rings N7–C8–C10–C17–C18 and C15–C19–C20–N16—C14–C13 being 3.4990 (16) Å.

## Database survey   

There are reports of structures with naphthalene-2,6-di­carboxyl­ate coordinating to Cu^II^ ions (Kanoo *et al.*, 2009 ▸; Zhao *et al.*, 2005 ▸; Gomez *et al.*, 2007 ▸; He *et al.*, 2005 ▸; Chen *et al.*, 2010 ▸) as well as compounds with the naphthalene-2,6-di­carboxyl­ate not coordinating (Tao *et al.*, 2003 ▸; Han *et al.*, 2012 ▸).

## Synthesis and crystallization   

Naphthalene-2,6-di­carb­oxy­lic acid (0.021 g, 0.10 mmol) was suspended in 10 ml of water; while stirring and heating, a concentrated solution of KOH was added until a transparent solution was obtained. A second solution was prepared by mixing 1,10-phenanthroline (0.018 g, 0.10 mmol) in MeOH (5 ml) and Cu(NO_3_)_2_·3H_2_0 (0.018 g, 0.21 mmol) dissolved in water (5 ml). Both solutions were mixed and stirred under reflux for a period of 3 h. The clear-blue solution was slowly evaporated at room temperature. Blue crystals of the title compound were obtained after several days. The yield was not determined due to the poor stability of the compound out of solution.

## Refinement   

Crystal data, data collection and crystal structure refinement details are summarized in Table 2 ▸. The hydroxide H and water H atoms were located in a difference Fourier map and positional parameters were refined with *U*_iso_(H) = 1.5*U*_eq_(O). Aromatic H atoms were placed in calculated positions and refined in riding mode, C—H = 0.93 Å and *U*_iso_(H) = 1.2*U*_eq_(C).

## Supplementary Material

Crystal structure: contains datablock(s) I, global. DOI: 10.1107/S2056989015004338/xu5835sup1.cif

Structure factors: contains datablock(s) I. DOI: 10.1107/S2056989015004338/xu5835Isup2.hkl

CCDC reference: 1051837

Additional supporting information:  crystallographic information; 3D view; checkCIF report

## Figures and Tables

**Table 1:** Hydrogen-bond geometry (, )

*D*H*A*	*D*H	H*A*	*D* *A*	*D*H*A*
O3H3*A*O5^i^	0.76(1)	2.24(2)	2.977(3)	166(3)
O4H4*A*O6	0.76(1)	2.07(2)	2.821(3)	168(4)
O4H4*B*O1^i^	0.76(1)	2.01(1)	2.769(3)	176(4)
O5H5*A*O1	0.76(1)	2.27(2)	2.993(3)	159(4)
O5H5*B*O2^ii^	0.76(1)	2.13(2)	2.846(3)	156(4)
O6H6*A*O1	0.76(1)	2.13(2)	2.882(3)	167(4)
O6H6*B*O7	0.77(1)	2.04(2)	2.782(4)	164(4)
O7H7*A*O3^iii^	0.76(1)	2.07(1)	2.820(3)	171(4)
O7H7*B*O2^iv^	0.76(1)	2.00(2)	2.744(3)	165(4)

Symmetry codes: (i) 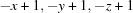; (ii) 
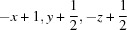
; (iii) 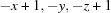; (iv) 
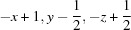
.

**Table 2:** Experimental details

Crystal data	
Chemical formula	[Cu_2_(OH)_2_(C_12_H_8_N_2_)_2_(H_2_O)_2_](C_12_H_6_O_4_)6H_2_O
*M*_r_	879.80
Crystal system, space group	Monoclinic, *P*2_1_/*c*
Temperature (K)	298
*a*, *b*, *c* ()	9.3626(16), 10.5812(18), 18.648(3)
()	100.961(3)
*V* (^3^)	1813.7(5)
*Z*	2
Radiation type	Mo *K*
(mm^1^)	1.25
Crystal size (mm)	0.32 0.14 0.13

Data collection	
Diffractometer	Bruker SMART *APEX* CCD
Absorption correction	Multi-scan (*SADABS*; Bruker, 2012 ▸)
*T*_min_, *T*_max_	0.691, 0.858
No. of measured, independent and observed [*I* > 2(*I*)] reflections	12102, 4168, 3164
*R*_int_	0.040
(sin /)_max_ (^1^)	0.651

Refinement	
*R*[*F* ^2^ > 2(*F* ^2^)], *wR*(*F* ^2^), *S*	0.037, 0.087, 1.03
No. of reflections	4168
No. of parameters	280
No. of restraints	36
H-atom treatment	H atoms treated by a mixture of independent and constrained refinement
_max_, _min_ (e ^3^)	0.41, 0.30

Computer programs: *APEX2* and *SAINT* (Bruker, 2012 ▸), *SHELXS2012* (Sheldrick, 2008 ▸), *SHELXL2014* (Sheldrick, 2015 ▸), *XP* in *SHELXTL* (Sheldrick, 2008 ▸) and *CIFTAB* (Sheldrick, 2013 ▸).

## supplementary crystallographic information

### Crystal data

**Table:**

[Cu_2_(OH)_2_(C_12_H_8_N_2_)_2_(H_2_O)_2_](C_12_H_6_O_4_)·6H_2_O	*F*(000) = 908
*M**_r_* = 879.80	*D*_x_ = 1.611 Mg m^−^^3^
Monoclinic, *P*2_1_/*c*	Mo *K*α radiation, λ = 0.71073 Å
*a* = 9.3626 (16) Å	Cell parameters from 4729 reflections
*b* = 10.5812 (18) Å	θ = 2.2–27.5°
*c* = 18.648 (3) Å	µ = 1.25 mm^−^^1^
β = 100.961 (3)°	*T* = 298 K
*V* = 1813.7 (5) Å^3^	Prism-hexagonal, blue
*Z* = 2	0.32 × 0.14 × 0.13 mm

### Data collection

**Table:**

Bruker SMART APEX CCD diffractometer	4168 independent reflections
Radiation source: fine-focus sealed tube	3164 reflections with *I* > 2σ(*I*)
Graphite monochromator	*R*_int_ = 0.040
Detector resolution: 8.333 pixels mm^-1^	θ_max_ = 27.6°, θ_min_ = 2.2°
φ and ω scans	*h* = −12→12
Absorption correction: multi-scan (*SADABS*; Bruker, 2012)	*k* = −13→13
*T*_min_ = 0.691, *T*_max_ = 0.858	*l* = −24→24
12102 measured reflections	

### Refinement

**Table:**

Refinement on *F*^2^	Primary atom site location: structure-invariant direct methods
Least-squares matrix: full	Secondary atom site location: difference Fourier map
*R*[*F*^2^ > 2σ(*F*^2^)] = 0.037	Hydrogen site location: inferred from neighbouring sites
*wR*(*F*^2^) = 0.087	H atoms treated by a mixture of independent and constrained refinement
*S* = 1.03	*w* = 1/[σ^2^(*F*_o_^2^) + (0.0327*P*)^2^ + 0.9957*P*] where *P* = (*F*_o_^2^ + 2*F*_c_^2^)/3
4168 reflections	(Δ/σ)_max_ = 0.001
280 parameters	Δρ_max_ = 0.41 e Å^−^^3^
36 restraints	Δρ_min_ = −0.30 e Å^−^^3^

### Special details

**Table:**

Geometry. All e.s.d.'s (except the e.s.d. in the dihedral angle between two l.s. planes) are estimated using the full covariance matrix. The cell e.s.d.'s are taken into account individually in the estimation of e.s.d.'s in distances, angles and torsion angles; correlations between e.s.d.'s in cell parameters are only used when they are defined by crystal symmetry. An approximate (isotropic) treatment of cell e.s.d.'s is used for estimating e.s.d.'s involving l.s. planes.

### Fractional atomic coordinates and isotropic or equivalent isotropic displacement parameters (Å^2^)

**Table:**

	*x*	*y*	*z*	*U*_iso_*/*U*_eq_	
Cu1	0.36968 (3)	0.07336 (3)	0.50127 (2)	0.02290 (10)	
O1	0.5472 (2)	0.60808 (19)	0.36776 (11)	0.0381 (5)	
O2	0.6367 (2)	0.5229 (2)	0.27719 (11)	0.0495 (6)	
O3	0.52734 (18)	−0.00259 (17)	0.57091 (9)	0.0261 (4)	
H3A	0.571 (3)	0.047 (2)	0.5945 (15)	0.039*	
O4	0.4830 (2)	0.26261 (19)	0.50689 (11)	0.0349 (5)	
H4A	0.456 (4)	0.303 (3)	0.4734 (12)	0.052*	
H4B	0.472 (4)	0.301 (3)	0.5401 (13)	0.052*	
O5	0.3358 (3)	0.8164 (2)	0.31710 (13)	0.0498 (6)	
H5A	0.387 (4)	0.760 (3)	0.319 (2)	0.075*	
H5B	0.341 (4)	0.855 (3)	0.2833 (14)	0.075*	
O6	0.3821 (3)	0.3788 (2)	0.37053 (15)	0.0525 (6)	
H6A	0.414 (4)	0.4451 (19)	0.371 (2)	0.079*	
H6B	0.428 (4)	0.333 (3)	0.352 (2)	0.079*	
O7	0.5308 (3)	0.1767 (2)	0.32376 (12)	0.0459 (6)	
H7A	0.519 (4)	0.135 (3)	0.3554 (15)	0.069*	
H7B	0.491 (4)	0.140 (3)	0.2907 (14)	0.069*	
C1	0.7904 (3)	0.5295 (2)	0.39346 (14)	0.0266 (6)	
C2	0.9135 (3)	0.4941 (3)	0.36397 (14)	0.0306 (6)	
H2	0.9045	0.4853	0.3137	0.037*	
C3	1.0446 (3)	0.4728 (3)	0.40805 (14)	0.0304 (6)	
H3	1.1242	0.4512	0.3874	0.036*	
C4	0.9383 (3)	0.5169 (2)	0.51493 (14)	0.0255 (5)	
C5	0.8052 (3)	0.5398 (2)	0.46729 (14)	0.0280 (6)	
H5	0.7247	0.5627	0.4869	0.034*	
C6	0.6480 (3)	0.5557 (3)	0.34251 (15)	0.0308 (6)	
N7	0.1858 (2)	0.11609 (19)	0.43037 (11)	0.0232 (5)	
C8	0.1602 (3)	0.1156 (3)	0.35799 (14)	0.0290 (6)	
H8	0.2362	0.0970	0.3341	0.035*	
C9	0.0233 (3)	0.1419 (3)	0.31650 (15)	0.0332 (6)	
H9	0.0093	0.1403	0.2658	0.040*	
C10	−0.0906 (3)	0.1702 (3)	0.35005 (15)	0.0320 (6)	
H10	−0.1821	0.1876	0.3225	0.038*	
C11	−0.1773 (3)	0.2011 (2)	0.46792 (16)	0.0314 (6)	
H11	−0.2711	0.2198	0.4435	0.038*	
C12	−0.1477 (3)	0.2013 (2)	0.54136 (15)	0.0304 (6)	
H12	−0.2214	0.2205	0.5667	0.037*	
C13	0.0330 (3)	0.1719 (3)	0.65763 (15)	0.0328 (6)	
H13	−0.0365	0.1890	0.6859	0.039*	
C14	0.1731 (3)	0.1457 (3)	0.69029 (15)	0.0341 (7)	
H14	0.1999	0.1467	0.7409	0.041*	
C15	0.2761 (3)	0.1174 (3)	0.64738 (14)	0.0290 (6)	
H15	0.3708	0.0985	0.6704	0.035*	
N16	0.2434 (2)	0.11657 (19)	0.57492 (11)	0.0230 (5)	
C17	0.0737 (2)	0.1444 (2)	0.46436 (14)	0.0217 (5)	
C18	−0.0673 (3)	0.1726 (2)	0.42647 (14)	0.0251 (5)	
C19	0.1051 (2)	0.1448 (2)	0.54237 (13)	0.0215 (5)	
C20	−0.0055 (3)	0.1728 (2)	0.58126 (14)	0.0256 (6)	

### Atomic displacement parameters (Å^2^)

**Table:**

	*U*^11^	*U*^22^	*U*^33^	*U*^12^	*U*^13^	*U*^23^
Cu1	0.01878 (16)	0.02928 (18)	0.02090 (16)	0.00393 (13)	0.00440 (11)	0.00009 (14)
O1	0.0302 (10)	0.0441 (12)	0.0373 (11)	0.0069 (9)	−0.0002 (9)	−0.0061 (9)
O2	0.0487 (13)	0.0674 (16)	0.0277 (11)	0.0166 (12)	−0.0042 (10)	−0.0074 (11)
O3	0.0238 (9)	0.0330 (11)	0.0208 (9)	0.0057 (8)	0.0022 (7)	−0.0031 (8)
O4	0.0370 (11)	0.0312 (12)	0.0372 (12)	0.0013 (9)	0.0087 (10)	−0.0018 (9)
O5	0.0523 (14)	0.0594 (17)	0.0414 (14)	0.0069 (12)	0.0186 (12)	0.0011 (12)
O6	0.0476 (14)	0.0491 (15)	0.0640 (16)	−0.0027 (12)	0.0191 (12)	0.0028 (14)
O7	0.0475 (13)	0.0484 (15)	0.0392 (14)	−0.0100 (11)	0.0014 (11)	0.0018 (11)
C1	0.0296 (14)	0.0209 (13)	0.0275 (14)	−0.0008 (11)	0.0008 (11)	0.0012 (11)
C2	0.0365 (15)	0.0322 (15)	0.0234 (13)	0.0000 (12)	0.0064 (11)	0.0002 (12)
C3	0.0314 (14)	0.0313 (15)	0.0298 (15)	0.0036 (12)	0.0089 (12)	−0.0016 (12)
C4	0.0285 (13)	0.0197 (13)	0.0281 (14)	0.0028 (11)	0.0052 (11)	−0.0008 (11)
C5	0.0296 (14)	0.0258 (14)	0.0298 (14)	0.0055 (11)	0.0090 (11)	−0.0011 (11)
C6	0.0349 (15)	0.0251 (14)	0.0306 (14)	0.0007 (12)	0.0016 (12)	0.0001 (12)
N7	0.0212 (10)	0.0248 (11)	0.0238 (11)	0.0006 (9)	0.0051 (9)	0.0010 (9)
C8	0.0284 (14)	0.0328 (15)	0.0263 (14)	0.0013 (11)	0.0069 (11)	0.0026 (12)
C9	0.0389 (16)	0.0334 (16)	0.0244 (14)	−0.0006 (13)	−0.0015 (12)	0.0050 (12)
C10	0.0265 (14)	0.0296 (15)	0.0355 (15)	0.0007 (12)	−0.0049 (12)	0.0039 (12)
C11	0.0179 (12)	0.0284 (15)	0.0468 (17)	0.0033 (11)	0.0032 (12)	−0.0039 (13)
C12	0.0229 (13)	0.0282 (15)	0.0425 (17)	0.0005 (11)	0.0122 (12)	−0.0039 (12)
C13	0.0340 (15)	0.0335 (15)	0.0348 (15)	−0.0025 (13)	0.0166 (12)	−0.0080 (13)
C14	0.0397 (16)	0.0379 (17)	0.0261 (14)	−0.0018 (13)	0.0096 (12)	−0.0050 (12)
C15	0.0284 (14)	0.0307 (15)	0.0266 (14)	−0.0011 (11)	0.0023 (11)	−0.0034 (11)
N16	0.0194 (10)	0.0239 (11)	0.0257 (11)	0.0003 (9)	0.0042 (9)	−0.0011 (9)
C17	0.0188 (12)	0.0171 (12)	0.0292 (13)	−0.0001 (10)	0.0047 (10)	0.0008 (10)
C18	0.0210 (12)	0.0195 (13)	0.0334 (14)	−0.0014 (10)	0.0012 (10)	0.0017 (11)
C19	0.0186 (12)	0.0195 (13)	0.0269 (13)	−0.0016 (10)	0.0053 (10)	−0.0018 (10)
C20	0.0233 (13)	0.0210 (13)	0.0344 (15)	−0.0030 (10)	0.0106 (11)	−0.0050 (11)

### Geometric parameters (Å, º)

**Table:**

Cu1—O3	1.9448 (17)	C4—C4^ii^	1.420 (5)
Cu1—O3^i^	1.9482 (17)	C5—H5	0.9300
Cu1—N7	2.012 (2)	N7—C8	1.325 (3)
Cu1—N16	2.028 (2)	N7—C17	1.358 (3)
Cu1—O4	2.259 (2)	C8—C9	1.394 (4)
Cu1—Cu1^i^	2.9002 (7)	C8—H8	0.9300
O1—C6	1.261 (3)	C9—C10	1.368 (4)
O2—C6	1.251 (3)	C9—H9	0.9300
O3—Cu1^i^	1.9481 (17)	C10—C18	1.400 (4)
O3—H3A	0.757 (13)	C10—H10	0.9300
O4—H4A	0.762 (13)	C11—C12	1.345 (4)
O4—H4B	0.762 (13)	C11—C18	1.432 (4)
O5—H5A	0.760 (13)	C11—H11	0.9300
O5—H5B	0.763 (13)	C12—C20	1.429 (3)
O6—H6A	0.763 (13)	C12—H12	0.9300
O6—H6B	0.766 (13)	C13—C14	1.365 (4)
O7—H7A	0.762 (13)	C13—C20	1.401 (4)
O7—H7B	0.761 (13)	C13—H13	0.9300
C1—C5	1.362 (4)	C14—C15	1.398 (4)
C1—C2	1.419 (4)	C14—H14	0.9300
C1—C6	1.508 (4)	C15—N16	1.328 (3)
C2—C3	1.359 (4)	C15—H15	0.9300
C2—H2	0.9300	N16—C19	1.355 (3)
C3—C4^ii^	1.419 (4)	C17—C18	1.406 (3)
C3—H3	0.9300	C17—C19	1.428 (3)
C4—C5	1.407 (4)	C19—C20	1.404 (3)
C4—C3^ii^	1.419 (4)		
			
O3—Cu1—O3^i^	83.69 (8)	C8—N7—C17	118.0 (2)
O3—Cu1—N7	167.78 (8)	C8—N7—Cu1	129.39 (17)
O3^i^—Cu1—N7	96.11 (8)	C17—N7—Cu1	112.53 (16)
O3—Cu1—N16	96.16 (8)	N7—C8—C9	122.3 (2)
O3^i^—Cu1—N16	169.61 (8)	N7—C8—H8	118.9
N7—Cu1—N16	81.84 (8)	C9—C8—H8	118.9
O3—Cu1—O4	92.59 (8)	C10—C9—C8	120.3 (3)
O3^i^—Cu1—O4	94.76 (8)	C10—C9—H9	119.9
N7—Cu1—O4	99.61 (8)	C8—C9—H9	119.9
N16—Cu1—O4	95.62 (8)	C9—C10—C18	119.0 (2)
O3—Cu1—Cu1^i^	41.89 (5)	C9—C10—H10	120.5
O3^i^—Cu1—Cu1^i^	41.80 (5)	C18—C10—H10	120.5
N7—Cu1—Cu1^i^	136.62 (6)	C12—C11—C18	121.3 (2)
N16—Cu1—Cu1^i^	137.11 (6)	C12—C11—H11	119.3
O4—Cu1—Cu1^i^	94.93 (6)	C18—C11—H11	119.3
Cu1—O3—Cu1^i^	96.31 (8)	C11—C12—C20	121.4 (2)
Cu1—O3—H3A	111 (2)	C11—C12—H12	119.3
Cu1^i^—O3—H3A	112 (2)	C20—C12—H12	119.3
Cu1—O4—H4A	112 (3)	C14—C13—C20	119.6 (2)
Cu1—O4—H4B	112 (3)	C14—C13—H13	120.2
H4A—O4—H4B	107 (4)	C20—C13—H13	120.2
H5A—O5—H5B	108 (4)	C13—C14—C15	119.8 (3)
H6A—O6—H6B	109 (4)	C13—C14—H14	120.1
H7A—O7—H7B	102 (4)	C15—C14—H14	120.1
C5—C1—C2	118.6 (2)	N16—C15—C14	122.2 (2)
C5—C1—C6	122.0 (2)	N16—C15—H15	118.9
C2—C1—C6	119.4 (2)	C14—C15—H15	118.9
C3—C2—C1	121.1 (2)	C15—N16—C19	118.1 (2)
C3—C2—H2	119.5	C15—N16—Cu1	129.74 (17)
C1—C2—H2	119.5	C19—N16—Cu1	112.16 (16)
C2—C3—C4^ii^	121.0 (2)	N7—C17—C18	123.2 (2)
C2—C3—H3	119.5	N7—C17—C19	116.7 (2)
C4^ii^—C3—H3	119.5	C18—C17—C19	120.2 (2)
C5—C4—C3^ii^	122.8 (2)	C10—C18—C17	117.2 (2)
C5—C4—C4^ii^	119.0 (3)	C10—C18—C11	124.3 (2)
C3^ii^—C4—C4^ii^	118.2 (3)	C17—C18—C11	118.4 (2)
C1—C5—C4	122.1 (2)	N16—C19—C20	123.4 (2)
C1—C5—H5	118.9	N16—C19—C17	116.7 (2)
C4—C5—H5	118.9	C20—C19—C17	119.9 (2)
O2—C6—O1	123.7 (3)	C13—C20—C19	116.8 (2)
O2—C6—C1	117.6 (2)	C13—C20—C12	124.4 (2)
O1—C6—C1	118.7 (2)	C19—C20—C12	118.8 (2)
			
C5—C1—C2—C3	−1.1 (4)	C9—C10—C18—C17	−0.5 (4)
C6—C1—C2—C3	178.3 (3)	C9—C10—C18—C11	179.9 (3)
C1—C2—C3—C4^ii^	1.1 (4)	N7—C17—C18—C10	0.3 (4)
C2—C1—C5—C4	0.2 (4)	C19—C17—C18—C10	−179.9 (2)
C6—C1—C5—C4	−179.2 (2)	N7—C17—C18—C11	−180.0 (2)
C3^ii^—C4—C5—C1	−180.0 (3)	C19—C17—C18—C11	−0.2 (4)
C4^ii^—C4—C5—C1	0.6 (5)	C12—C11—C18—C10	179.8 (3)
C5—C1—C6—O2	−167.8 (3)	C12—C11—C18—C17	0.1 (4)
C2—C1—C6—O2	12.9 (4)	C15—N16—C19—C20	−1.1 (4)
C5—C1—C6—O1	11.8 (4)	Cu1—N16—C19—C20	177.23 (19)
C2—C1—C6—O1	−167.6 (3)	C15—N16—C19—C17	179.4 (2)
C17—N7—C8—C9	−0.5 (4)	Cu1—N16—C19—C17	−2.3 (3)
Cu1—N7—C8—C9	176.7 (2)	N7—C17—C19—N16	−0.2 (3)
N7—C8—C9—C10	0.3 (4)	C18—C17—C19—N16	179.9 (2)
C8—C9—C10—C18	0.2 (4)	N7—C17—C19—C20	−179.8 (2)
C18—C11—C12—C20	−0.2 (4)	C18—C17—C19—C20	0.4 (4)
C20—C13—C14—C15	−1.4 (4)	C14—C13—C20—C19	0.6 (4)
C13—C14—C15—N16	1.0 (4)	C14—C13—C20—C12	−178.7 (3)
C14—C15—N16—C19	0.3 (4)	N16—C19—C20—C13	0.6 (4)
C14—C15—N16—Cu1	−177.7 (2)	C17—C19—C20—C13	−179.8 (2)
C8—N7—C17—C18	0.2 (4)	N16—C19—C20—C12	180.0 (2)
Cu1—N7—C17—C18	−177.50 (19)	C17—C19—C20—C12	−0.5 (4)
C8—N7—C17—C19	−179.7 (2)	C11—C12—C20—C13	179.7 (3)
Cu1—N7—C17—C19	2.7 (3)	C11—C12—C20—C19	0.4 (4)

Symmetry codes: (i) −*x*+1, −*y*, −*z*+1; (ii) −*x*+2, −*y*+1, −*z*+1.

### Hydrogen-bond geometry (Å, º)

**Table:**

*D*—H···*A*	*D*—H	H···*A*	*D*···*A*	*D*—H···*A*
O3—H3*A*···O5^iii^	0.76 (1)	2.24 (2)	2.977 (3)	166 (3)
O4—H4*A*···O6	0.76 (1)	2.07 (2)	2.821 (3)	168 (4)
O4—H4*B*···O1^iii^	0.76 (1)	2.01 (1)	2.769 (3)	176 (4)
O5—H5*A*···O1	0.76 (1)	2.27 (2)	2.993 (3)	159 (4)
O5—H5*B*···O2^iv^	0.76 (1)	2.13 (2)	2.846 (3)	156 (4)
O6—H6*A*···O1	0.76 (1)	2.13 (2)	2.882 (3)	167 (4)
O6—H6*B*···O7	0.77 (1)	2.04 (2)	2.782 (4)	164 (4)
O7—H7*A*···O3^i^	0.76 (1)	2.07 (1)	2.820 (3)	171 (4)
O7—H7*B*···O2^v^	0.76 (1)	2.00 (2)	2.744 (3)	165 (4)

Symmetry codes: (i) −*x*+1, −*y*, −*z*+1; (iii) −*x*+1, −*y*+1, −*z*+1; (iv) −*x*+1, *y*+1/2, −*z*+1/2; (v) −*x*+1, *y*−1/2, −*z*+1/2.
